# Impact of perinatal different intrauterine environments on child growth and development in the first six months of life - IVAPSA birth cohort: rationale, design, and methods

**DOI:** 10.1186/1471-2393-12-25

**Published:** 2012-04-02

**Authors:** Juliana Rombaldi Bernardi, Charles Francisco Ferreira, Marina Nunes, Clécio Homrich da Silva, Vera Lúcia Bosa, Patrícia Pelufo Silveira, Marcelo Zubaran Goldani

**Affiliations:** 1 Núcleo de Estudos da Saúde da Criança e do Adolescente – Hospital de Clínicas de Porto Alegre – Faculdade de Medicina - Universidade Federal do Rio Grande do Sul. Rua Ramiro Barcelos, 2350, CEP 90035-903 - Porto Alegre/RS – Brazil

**Keywords:** Infant, Low birth weight, Preterm birth, DOHaD, Programming, Barker hypothesis

## Abstract

**Background:**

In the last twenty years, retrospective studies have shown that perinatal events may impact the individual health in the medium and long term. However, only a few prospective studies were designed to address this phenomenon. This study aims to describe the design and methods of the Impact of Perinatal Environmental Variations in the First Six Months of Life - the IVAPSA Birth Cohort.

**Method/Design:**

This is a clinical study and involves the recruitment of a birth cohort from hospitals in Porto Alegre, Rio Grande do Sul, Brazil. Mothers from different clinical backgrounds (hypertensive, diabetics, smokers, having an intrauterine growth restricted child for idiopathic reasons, and controls) will be invited to join the study twenty-four hours after the birth of their child. Data on economic, social, and maternal health care, feeding practices, anthropometric measures, physical activity, and neuropsychological evaluation will be obtained in interviews at postpartum, 7 and 15 days, 1, 3 and 6 months of life.

**Discussion:**

To our knowledge, this is the first thematic cohort focused on the effects of intrauterine growth restriction to prospectively enroll mothers from different clinical backgrounds. The IVAPSA Birth Cohort is a promising research platform that can contribute to the knowledge on the relationship between perinatal events and their consequences on the children's early life.

## Background

Over the past twenty years, several studies have shown that perinatal events may impact the individual's health in the medium and long term. The initial reports of Barker and colleagues, relating low birth weight with increased cardiovascular risk in adulthood, were followed by studies showing that, as a group, low birth weight subjects persisted biologically different from those of adequate weight until adulthood. They had higher blood pressure [1], being more likely to develop type II diabetes [2] and metabolic syndrome as adults [3]. In addition, in subsequent reports, these and other researchers demonstrated that low birth weight was associated with an altered pattern of plasma lipids [4], reduced bone density [5] differentiated responses to stress [6], less elastic arteries [7], specific patterns of hormone secretion [8] and higher incidence of depression [9,10]. Moreover, it has been shown that different insults during the pregnancy and neonatal period bring long-term consequences to the offspring, even without affecting birth weight [11,12]. In this protocol, we aim to address these ideas, grouped into a new branch of scientific knowledge called "Developmental Origins of Health and Disease" (DOHaD).

To date, most of these studies were performed 1) in preexisting birth cohorts, in which the primary study hypothesis were not related to the DOHaD ideas [13-17] and 2) in developed countries. Only a few prospective birth cohorts were designed after the DOHaD concepts arose [18-23], most of them focusing on low birth weight as the main independent variable. However, many studies are demonstrating that rather than birth weight, the maternal phenotype in which the fetus will develop (maternal behavior, metabolism, drug use and diseases) is more important to determine the future child's health and disease patterns [24-31]. These new evidence prompted our research group to design this project - Impact of Perinatal Environmental Variations in the First Six Months of Life - the IVAPSA Birth Cohort.

Recent surveys support these ideas by demonstrating interactions between the environment and gene expression at different levels. Not only the cellular environment affects gene expression and protein production, but the individual's relationships with the environment may also influence aspects of behavior, morphology and gene expression, even in a matter of hours [32]. Important studies show that the influence of interactions occurred at vulnerable or susceptible periods to programming, through epigenetic effects, may persist even in a transgenerational way [33,34]. However, there are no studies to assess the interaction between changes in the intrauterine environment determinants of intrauterine growth restriction (IUGR) and the individual genetic characteristics on growth and long-term health. For example, it is unclear whether individuals born with IUGR due to exposure to smoking during pregnancy will have the same health and disease pattern as individuals born IUGR due to maternal malnutrition or other factors will. Besides, these two IUGR subtypes may interact differently with the subject's genotype to affect its health and disease patterns.

Our group is interested in understanding the long-term effects of these fetal environmental changes on the offspring's' growth, behavior, metabolism and neurodevelopment, as well as in the early identification of vulnerability to the deleterious effects of these changes. Thus, this project aims to assess the impact of variations in the perinatal environment on the children's health in their first six months of life.

The aims of this longitudinal study are divided in four domains:

1. Nutritional

There is an association between the IUGR infant and acceleration growth in early postnatal life and the emergence of insulin resistance, visceral obesity and glucose intolerance in adult life [35]. In addition, it is known that about 10% of IUGR children will never catch up in growth, a condition that may have an impact in the child's metabolism and later risk for disease [36]. In this study we intend to investigate whether children born IUGR for different reasons grow differently. Besides, we intend to verify interactions between these IUGR types with environmental variations (nutrition, breast milk composition, maternal behavior), aiming at identifying early vulnerability for growth failure or obesity risk and its related metabolic consequences.

2. Behavioral

Studies from our group [37,30] and others [38,39] have shown that the IUGR and smoking during gestation are associated with changes in eating behavior in the long term, increasing the preference for palatable foods. This altered relationship with food cues is identifiable already early in life [40]. Besides, we [41] and others [42-44] have shown that low birth weight leads to increased vulnerability to sedentary behavior, in a complex interaction with environmental variables such as SES (socioeconomic status) [41]. Therefore it would be of extreme importance to investigate if different IUGR profiles would lead to differential patterns of vulnerability to obesity by altering food practices or the willing to exercise from early stages in life.

3. Molecular

Is it known that there is an interaction between genotype and size at birth on the risk for diabetes [45]; moreover other studies have shown interaction between polymorphic variations and perinatal environment on risk for disease in adulthood [46,47] reflecting a gene-environment interactions. However, there are only a few studies investigating how variations in maternal phenotype interact with the genotype of mother-child pairs and impact child growth and health.

4. Neurodevelopment

Evidence suggests that there are adverse effects of being born IUGR on the functioning and development of the offspring, affecting the cognitive development [48,49], academic achievement/professional attainment [50] and the risk for psychopathology in later life [51,52]. Children who are exposed to malnutrition early in life may experience lasting effects on their brain architecture and persistent disruptions of their stress response systems [6,53]. However, only a few studies investigate the interaction between IUGR and environmental variations such as nutrition on these outcomes [54]. Besides that, it is not known if different IUGR profiles derived from the various maternal phenotypes during gestation (maternal smoking, hypertension, etc.) or right after birth (maternal care, depression, etc.) would have a differential vulnerability to these neurodevelopment alterations.

## Methods/Design

**Figure 1:**
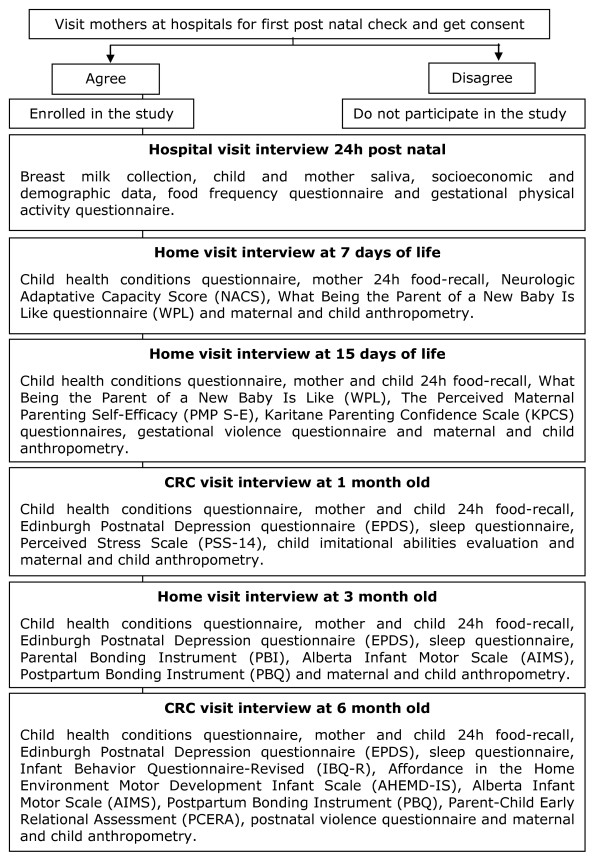
**Flow diagram of the recruitment strategy for the cohort study and data collection procedures**.

This is a thematic, prospective, longitudinal birth cohort with the aim to assess the interactions between the maternal phenotype during gestation (maternal smoking, hypertension, diabetes), the maternal/fetal genotype and their associations with outcomes related to growth, behavior and neurodevelopment (Figure 1).

### Participants

Postpartum women assisted by Grupo Hospitalar Conceição (GHC) and Hospital de Clínicas de Porto Alegre (HCPA) residing in Porto Alegre, Rio Grande do Sul, Brazil. Exclusion criteria are HIV patients, and newborn from twin gestations, having malformations or chronic disease at birth or requiring hospitalization.

### Outcome measures

The proposed primary and secondary outcome measures are shown in Table 1.

**Table 1:** Outcome measures for the cohort study

Outcome	Parental outcomes	Child outcomes
1. Nutritional	FFQ	Questions about breastfeeding and
1.1 Dietary Data	24 h-recalls -	complementary feeding 24 h-recalls

1.2 Physical activity	IPAQ short form	-

1.3 Antropometry	Body weight	Birth data
	Height	Body weight
	Arm circumference	Length
	Subscapular skinfold	Arm circumference
	Waist circumference	Head circumference
	Triceps skinfold	Subscapular skinfold
		Triceps skinfold

2. Behavior	WPL	IBQ-R
	PMP S-E	PCERA
	KPCS	-
	PSS-14	-
	PBI	-
	PBQ	-
	EPDS	-
	Domestic Violence	-

3. Neurodevelopment	-	NACS
	-	AIMS
	-	AHEMD-IS
	- -	Child imitational abilities evaluation

4. Molecular	Breast milk	DNA
	DNA	-

### General information

Interviews will be performed at home or at the clinical research center (CRC) in located at the Hospital de Clínicas de Porto Alegre (HCPA). During the interviews, familial demographic, socioeconomic, and environmental variables will be investigated. The ABEP (Brazilian Researching Companies Association) questionnaire [55], an official governmental document that assesses the economic status for the Brazilian population, will be applied. Mothers will be evaluated regarding the obstetric and medical history, the use of medications use and smoking habits. Children will be evaluated using the birth data collected from the charts (date of birth, sex, age, weight and length, Apgar score and complications), as well as with questions about breastfeeding, complementary feeding and a questionnaire about sleep behavior.

### Team training and manual

During the first step of the study, the researchers will be received training weekly during one year, based on questionnaires, protocols and anthropometrics measures, and invited experts in the area: psychiatrist, neurologist and pediatrics. A training manual will be used to ensure standardization of instructions and consistency of training standards for project workers throughout the life of the project.

### Data collections instruments according to the four domains

#### Nutritional

##### Dietary data

Food intake estimate will be assessed by 24 h recall applied for both mother and child in different occasions and by an 88-itens Food Frequency Questionnaire (FFQ), validated for the pregnant women in Brazil [56]. The quantitative analysis of macronutrients and micronutrients consumed will be calculated with the use of NutriBase^® ^*software *(Versão NB7 Network) [Phoenix, AZ, USD].

##### Physical activity

Physical activity during pregnancy will be assessed by the International Physical Activity Questionnaire (IPAQ) short form. The items in the IPAQ short form were structured to provide separate scores on walking, moderate-intensity and vigorous-intensity activity [57]. This instrument is proposed by the World Health Organization (WHO) to determine physical activity levels, and was used in different studies having a good correlation with actual activity levels [58].

##### Anthropometry

Anthropometric measurements will be measured in duplicate to ensure minimum inter- and intra-observer variability, and taken through the use of standard techniques and calibrated equipment.

Body weight of the mothers and infants will be measured in kilograms with a portable digital electronic scale (Marte^®^) accurate to the nearest 50 g, wearing minimal clothing and without shoes. The infants will be weight in the arms of mothers, for the scale tare. The infant's weight will be then calculated by subtracting the total body weight of the mother from the weight of both mother and child.

Stature of mothers and infants will be measured with an extensible portable stadiometer (Alturexata^®^). The infant's recumbent length will be measured in a supine position, placed on a flat, stable surface such as a table. The mother height will be measured using a height board mounted at a right angle between a level floor and against a straight, vertical surface such as a wall or pillar.

Weight in kilograms and height in meters when used to calculate body mass index (BMI). Body weight and a weight gain of the pregnant women will be reported from the charts. The gestational weight gain and prepregnancy BMI will be classified according to the recommendations of the Institute of Medicine [59]. The nutritional status of infants will be evaluated using age specific values of height, weight and BMI using the World Health Organization [60] growth chart.

Arm circumference, head and waist circumference will be measured with an inextensible tape measure. The arm circumference of mothers and infants will be measured always at the left arm at the medium point between the acromium and olecranum. Head circumference of infants will be measured at the largest occipitofrontal circumference. The waist circumference of mothers will be measured midway between the costal margin and iliac crest.

The subscapular and triceps skinfolds will be assessed using a caliper (Lange^®^). In infants skinfold thickness will be measured starting at 3 months. Triceps skinfold of mothers and infants will be measured at the same levels as the arm circumference and subscapular skinfold will be measured and at the lower angle of the scapula, in the axis of the skin crease.

The arm and head circumference, subscapular and triceps skinfolds of infants will be evaluated using age specific using the World Health Organization [61] growth chart.

#### Questionnaires assessing behavior

The following instruments will be applied to assess parental care: the What Being the Parent of a New Baby Is Like (WPL) [62]; the Perceived Maternal Parenting Self-Efficacy (PMP S-E) [63] and the Karitane Parenting Confidence Scale (KPCS) [64] at the home visit interview at 15 days of life. These protocols will be translated and validated to Brazilian Portuguese during the course of team training.

The WPL was developed to examine parents' perceptions of themselves as parents and of the parenting experience with young infants, and was revised by improving its subscales: Success, Centrality and Life Change [62]. It consists of 25 items on a 9-point graphic rating scale. The end points of each scale were labeled with a bipolar descriptor, for example, "not at all difficult" and "very difficult". It was used in study verifying maternal satisfaction scores [65].

The PMP S-E tool, which is made up of 20 items representing four theorized subscales, was developed to assess mothers' perceptions of their ability to parent (maternal parenting self-efficacy) [63]. In this instrument, the mother will choose if the 'strongly disagrees' (score 1) to 'strongly agrees' (score 4) with the situation described. A low score on this scale indicates a low maternal self-efficacy. It was used in study verifying maternal social support and maternal parental self-efficacy with postnatal depression at 6 weeks post delivery [66].

The KPCS tool, a 15-item perceived parental self-efficacy measure, is useful clinically in screening for parenting difficulties, targeting interventions and evaluating outcomes [64]. In this instrument, the mother will choose if the situations described are true or not at all, or at which frequency they are true, by choosing to answer "no", "hardly ever"; "no, not very often"; "yes, some of the time"; and "yes, most of the time". It was used in study assessing the overall construct of parenting confidence and the correlated to Being a Mother scale (BaM-13) [67].

The Perceived Stress Scale (PSS-14) will be applied when child is 1 month old. The instrument is composed by 14 questions that were designed to check how unpredictable, uncontrollable and overloaded respondents evaluate their lives [68]. This instrument has questions with response options that range from zero to four (0 = never; 1 = almost never; 2 = sometimes; 3 = often and 4 = always). It was used in studies addressing perceived stress, which assesses the extent to which a respondent considers life situations to be stressful, and the association with food consumption [69].

The Parental Bonding Instrument (PBI) is composed by 25 questions in which the respondent answers in relation to paternal or maternal behavior until his 16 years of age [70,71]. The instrument will be applied when the child is 3 months of life. It is classically used to address parental behavior in two subscales: warmth and overprotection, showing high association with the individual's depression and anxiety in adult life [72]. Because parental care shows some stability throughout generations [73] this instrument could potentially predict the parental care that the child will be raised on during her childhood [74].

The Infant Behavior Questionnaire-Revised (IBQ-R) is composed of 184 items that assess the following the following 14 dimensions of temperament in infant: Activity Level, Distress to Limitations, Approach, Fear, Duration of Orienting, Smiling and Laughter, Vocal Reactivity, Sadness, Perceptual Sensitivity, High Intensity Pleasure, Low Intensity Pleasure, Cuddliness, Soothability, and Falling Reactivity. Parents will be asked to rate the frequency of specific temperament-related behaviors observed over the past week (or sometimes 2 weeks) in an ordinal scale ranging from 1 (never) to 7 (always) [75]. The instrument will be applied when child is 6 months of life. It was used in studies examining the relationship between breastfeeding and maternally-rated infant temperament [76].

The Parental Bonding Questionnaire (PBQ) is a screening instrument specifically targeted at disorders of the early mother-infant relationship. It is composed by 25 questions, in which the mother will choose responses to each item on a 6-point Likert scale, with the scale points labelled "always", "very often", "quite often", "sometimes", "rarely" and "never" [77]. It was used in studies to screen for mother-infant relationship disorders and to assess the severity of the disorders [78].

Postpartum depression is the most common complication of childbearing. The 10-question Edinburgh Postnatal Depression Scale (EPDS) is a valuable and efficient way of identifying women at risk for perinatal depression [79]. The scale indicates how the mother has felt during the past seven days and the response format is frequency-based. The responses to the 10 items are added together to obtain a score. It was used in studies assessing the prevalence and the background factors of maternal depressive symptoms and their relation to the quality of mother-infant interaction in a group of preterm infants and their mothers and the number of postnatal signs of depression was associated negatively with the quality of the maternal interaction behaviour with their preterm infants [80].

To know if mothers suffered domestic violence, a specific questionnaire will be applied: Abuse Assessment Screen [81,82] which has already been used and published by a group of Porto Alegre, Brazil [83]. This protocol is divided into four parts: oral aggressions, physical aggressions, aggressions with gun, knife or other and sexual abuse. It will be applied in two moments and will assess violence during gestation and post gestational violence.

The Parent-child Early Relational Assessment (PCERA) was designed to measure the quality of affect and behavior in parent-child interactions [84]. The instrument uses ratings that are based on observations of 5-minute videotaped interactions, including feeding, structured task, and free play. The PCERA will be applied at 6 months visit.

#### Neurodevelopment

The Neurologic Adaptive Capacity Score (NACS) was developed to detect central nervous system depression in term neonates exposed to intrapartum medications [85,86]. After that, the testing was used in numerous other studies as a form of assessment of neurological status of the newborn, with good correlation with long term neurodevelopmental assessment [87]. It consists of 20 items arranged into two subscales: adaptive capacity and neurologic evaluation. The neurologic subscale is further divided into four parts testing passive tone, active tone, primary reflexes, and general neurologic status. Each of the 20 items is assigned 0, 1, or 2 points depending on the infant's response. The maximum total score possible is 40. The NACS will be applied at the 7 days visit.

The Alberta Infant Motor Scale (AIMS) is a standardized scale that intends to evaluate and monitor the gross motor function in infants, by means of spontaneous motor activity observation from birth to 18 months or until the child learns to walk. It was developed to assess infants at risk of developing neuromotor dysfunctions. It quantifies gross motor activity in a global score, taking into consideration three criteria related to the quality of movement: weight distribution, posture and movement against the force of gravity [88,89]. It has a good correlation with the final gross motor abilities attained in later childhood [90]. The AIMS will be applied at the 3 and 6 months visit.

The Affordances in the Home Environment for Motor Development - Infant Scale (AHEMD-IS) will be applied at 6 months of life. It aims to assess the quantity and quality of affordances in the home environment that are conducive to motor development for infants 3- to 18 months. The structure for the AHEMD-IS was founded on the same 5-factor model 1 proposed in the original AHEMD [91]. As with the AHEMD, three types of tests were determined acceptable: simple dichotomic choice, 4-point Likert-type scale, and description-based queries. In addition, pictorial examples of the general classification are provided to help parents identify available categories and specific items. The AHEMD scores were shown to be associated with motor development in several studies [92].

The presence and the nature of hand fingers and facial movement imitation will be examined in infants [93,94]. The instrument uses ratings that are based on observations of 6-minute videotaped. The child imitational abilities evaluation will be applied at 1 month visit. Study of mirror-neurons systems is of functional importance to individual's role in the development of imitation [95].

#### Biological samples

##### Breast milk collection and storage for milk composition and hormones

The women will be advised to wash their hands with soap and water before milking and washing their breasts with water [96,97]. The collection procedure will be performed by the mothers, under appropriate supervision provided by the researcher. Milk was collected, under supervision, by manual expression directly into a plastic recipient. To express breast milk by hand mothers or researchers will be have to massage in the breast by starting at the top of the breast in circular manner with the C shape position. After collection, samples will be properly transported and stored at -80°C at the Translational Pediatrics Laboratory until analysis.

Fatty acids will be determined in phospholipids, triglyceride, and cholesterol ester fractions. Lipids will be extracted from serum with chloroform-methanol (2:1 by volume) according to the method of Folch et al., 1957 [98]. Fatty acid fractions will be separated by thin-layer chromatography using a silica gel plate and mobile-phase development, using a mixture of hexane, diethyl ether, and acetic acid glacial [99]. Fractions will be visualized by iodine vapor. Phospholipids, triglyceride, and cholesterol ester bands were scraped into separate tubes, and lipids were extracted from silica with chloroform-methanol and converted into fatty acid methyl esters by boron trifluoride catalysis [100]. The methyl esters will be then separated and measured by gas chromatography on specific capillary column. Analysis was performed on a Hewlett-Packard 6890 gas chromatograph equipped with a flame ionization detector. Helium will be used as carrier gas and nitrogen as make-up gas. The injection port temperature will be 200°C and the detector temperature 250°C. The column temperature will be held at 160°C for 5 min and increased to 190°C at a rate of 2°C/min; it was then held at this temperature for 2 min, and increased again to 220°C at a rate of 1°C/min. The identity of each fatty acid peak will be ascertained by comparison of peak retention time with a previously characterized mixture of 20 fatty acids. The relative amount of each fatty acid (% of total fatty acid) will be quantified by integrating the area under the peak and dividing the result by the total area for all fatty acids.

Total carbohydrates from milk will be determined by phenol-sulfuric acid assay, using the [101] method in a microplate format. Protein concentration will be quantified using bicinchoninic acid (BCA) protein assay. Milk leptin and corticosterone levels will be analyzed by ELISA using commercial kits.

##### DNA extraction and genotyping

The DNA will be extracted from saliva samples collected from mothers and their children at the postpartum interview using the Isohelix DNA Buccal Swabs^® ^(SK-2, Isohelix, United Kingdon). Mothers will be instructed to first rinse with 100 ml of distilled water and the collection is made by scraping the inner face of the cheeks with sterile cytological brushes, with circular movements repeated about 30 times. Then the brushes have their outer portion of the stems cut and placed in 2 ml microtubes. The samples will be stored in the refrigerator for a period of 2 to 30 days before extraction.

For the DNA extraction, 200 μl TES (10 mM Tris HCl, pH 7.6, 1 mM EDTA, 0.6% SDS) and 5 μl of proteinase K (10 mg/mL) are added to the tubes containing the swab and incubated for 2 h at 42 C. Then, the brush is removed and it is added 42 μl of saturated NaCl (6 M), stirring by hand with vigor. The sample will be centrifuged for 1 minute at 15,000 g, the supernatant transferred to a new tube and added 2 times the volume of absolute ethanol. The tubes are agitated and centrifuged for 1 minute at 15,000 g. The pure ethanol is discarded and will be added 1 ml of 70% ethanol by inverting the tubes several times to wash the pellet. Then tubes are centrifuged again for 1 minute at 15,000 g and the supernatant is discarded. Rinsing with 70% ethanol is repeated and, after discarding the supernatant, the tubes remain open for 30 minutes to evaporate residual ethanol. The DNA is dissolved in 60 μl TE 10:0,1 (10 mM Tris HCl, 0.1 mMEDTA).

##### Leptin Gene and Leptin Receptor

Proopiomelanocortin gene (Pomc), Fat Mass- and Obesity-Associated gene (FTO, rs9939609 gene containing the risk A allele) and Dopamine receptor D4 gene (Drd4) expression will be measured by real-time quantitative reverse transcription-polymerase chain reaction (RT-PCR) using an inventoried TaqMan FAM/MGB assay (Applied Biosystems). Expression values will be normalized by GAPD endogenous control expression using a TaqMan VIC/MGB endogenous control inventoried assay (Applied Biosystems, 4352340E). Quantification will be carried out by Nanodrop. Reactions will be performed in ABI Prism 7500 sequence detection instrument, which directly detects the RT-PCR product without downstream processing. Reactions will be carried out in a total volume of 12 ml containing 6 ml of 2x TaqMan Gene Expression Master Mix (containing ROX, Amplitaq Gold DNA polimerase, AmpErase UNG, dATP, dCTP, dGTP, dUTP, and MgCl2), 0.6 ml of 20x TaqMan Gene Expression Assay, 0.6 ml of 20x TaqMan Endegenous Control, 3.8 ml of water and 1 ml of DNA solution. Cycling program consists of 2 min at 50°C and 10 min at 95°C, followed by 40 cycles of 15 s at 95°C and 1 min at 60°C. All reactions will be performed in triplicates. Relative expression levels will be determined by the ddCt method [101,102].

### Sample size

Based on the effect size of 0.5 standard deviation (SD) of difference between the average Z-score of weight at 12 months of age, level of significance of 5% and test power of 80%, was calculated 72 mother-child pairs per group and 144 pairs in the control group, summing up a total 432 pairs calculating a loss to follow up of 20%, the size of the final sample consists of 521 mother-child pairs.

### Data analyses

Analysis will be conducted at the individual level using Statistical Package for Social Sciences (SPSS) version 18.0. It will be performed a descriptive analysis of continuous and categorical variables. The parametric data will be expressed as mean ± standard deviation. To detect the difference between variables with normal distribution, it will be used the ANOVA/ANCOVA tests followed by Bonferroni post hoc. The chi-square will be used to detect differences in proportions between groups. Significance level of 5% (*p *< 0.05) and 95% range will be considered.

### Ethical considerations

Ethical approval to conduct this study has been granted by the Ethics Committee of Hospital de Clínicas de Porto Alegre (HCPA) with the number 11-0097 and Ethics Committee of Grupo Hospitalar Conceição (GHC) with the number 11-027. After the eligibility criteria are met, postpartum women will be invited to enter the study, and only those that provide a written consent will be included.

### Financial support

The research will be supported by National Support Program for Centers of Excellence PRONEX 2009; FAPERGS/CNPq 10/0018.3 and FIPE/HCPA (Fundo de Incentivo à Pesquisa e Eventos do Hospital de Clínicas de Porto Alegre).

## Discussion

To our knowledge, this is the first prospective cohort to incorporate different maternal phenotypes in terms of pathological states to provide evidence of impact in developmental origins of health and disease.

Epidemiological studies in different parts of the world relate to the influence of certain environmental factors in early life with changes in the genetic expression, determining a peculiar profile of health and disease. Also, clinical and preclinical research point out to in the same direction, suggesting a strong association between environmental damages that occurred during the fetal period or in the early stages of extrauterine life with the emergence of chronic diseases throughout life. These findings provide new links of causality, inferring the possibility of building early behavioral, neurochemical and metabolic adjustments determinants of morbid outcomes in the long term.

Future perspectives for the IVAPSA Birth Cohort include the follow up these children until 5 years at least. The IVAPSA Project is a promising research platform that can contribute to the knowledge on the relationship between perinatal events and their consequences on children's and adult health.

## Abbreviations

AHEMD-IS: Affordances in the home environment for motor development-infant scale; AIMS: Alberta infant motor scale; BaM: Being a mother scale; BCA: Bicinchoninic acid; BMI: Body mass index; CRC: Clinical research center; DOHaD: Development origins of health and disease; EPDS: Edinburgh postnatal depression scale; FFQ: Food frequency questionnaire; GHC: Grupo Hospitalar Conceição; HCPA: Hospital de Clínicas de Porto Alegre; IBQ-R: Infant behavior questionnaire-revised; IUGR: Intrauterine growth restriction; IPAQ: International physical activity questionnaire; IVAPSA: Impact of perinatal environmental variations in the first six month of life; KPCS: Katarine parenting confidence scale; NACS: Neurologic adaptive capacity score; PBI: Parental bonding instrument; PBQ: Parental bonding questionnaire; PCERA: Parent-child early relational assessment; PMP S-E: Perceived maternal parenting self-efficacy; PSS-14: Perceived stress scale; SES: Socioeconomic status; SD: Standard deviation; SPSS: Statistical package for social sciences; WPL: What being the parent of a new baby is like; WHO: World Health Organization

## Competing interests

The authors declare that they have no competing interests.

## Authors' contributions

JRB was responsible for the data collection, executed the IVAPSA study and drafted the manuscript. CFF executed the IVAPSA study and drafted the manuscript. MN was responsible for the data collection, executed the IVAPSA study and drafted the manuscript. VLB supervised the IVAPSA study. CH da S supervised the IVAPSA study. PPS was responsible for the study design, supervised the IVAPSA study and drafted the manuscript. MZG was responsible for the study design, coordinated the IVAPSA study and drafted the manuscript. IVAPSA group were responsible for the data collection and executed the IVAPSA study. All authors meet the criteria for authorship and have read and approved the final manuscript.

## Authors' information

^1 ^Núcleo de Estudos da Saúde da Criança e do Adolescente, Faculdade de Medicina, Universidade Federal do Rio Grande do Sul, Porto Alegre, Brazil.

## Pre-publication history

The pre-publication history for this paper can be accessed here:

http://www.biomedcentral.com/1471-2393/12/25/prepub
